# Investigation of Solution Microstructure in Ferric Sulfate Coagulation-Assisted Precipitation of Fluoride Ions

**DOI:** 10.3390/molecules30061362

**Published:** 2025-03-18

**Authors:** Haodong Chen, Caocheng Li, Yuefei Zhang, Wen Fang, Lian Zou, Ruan Chi

**Affiliations:** 1 Key Laboratory for Green Chemical Process of Ministry of Education, Wuhan Institute of Technology, Wuhan 430205, China; 22209010014@stu.wit.edu.cn (H.C.); 22309010033@stu.wit.edu.cn (C.L.); racwit@163.com (R.C.); 2 School of Chemistry and Environmental Engineering, Wuhan Institute of Technology, Wuhan 430205, China; fangwen@jhun.edu.cn; 3 School of Electronic Information, Wuhan University, Wuhan 430072, China; zoulian@whu.edu.cn; 4 School of Xingfa Mining Engineering, Wuhan Institute of Technology, Wuhan 430073, China

**Keywords:** coagulation, fluoride, hydrated clusters, density functional theory, molecular dynamics

## Abstract

The solution microstructure during the ferric sulfate-assisted precipitation of calcium fluoride was systematically investigated using molecular dynamics simulations and DFT methods. The microscopic behavior of various ions in a calcium fluoride box in the presence of ferric sulfate was simulated using MD. The corresponding hydrated cluster structures were extracted from the MD trajectory; then, the structure was optimized and the frequency was calculated at the B3LYP/6–311++G(d, p) level. The results show that no hydrated clusters had imaginary frequencies. Based on the topology, interaction region indicator, and surface electrostatic potential and binding energy analysis of the hydrated clusters, it was revealed that ferric ions are easily hydrolyzed to form hydrated clusters of ferric hydroxide at higher pH levels. The most stable of these structures is [Fe(OH)_3_·(H_2_O)_2_], which has the lowest binding energy. During the ferric sulfate coagulation process, calcium fluoride clusters and ferric hydroxide clusters could form binuclear clusters through electrostatic interaction. The two metal centers in the binuclear cluster, Ca and Fe, are connected by hydroxide ions.

## 1. Introduction

High-concentration fluoride-containing wastewater primarily comes from the phosphate fertilizer industry, aluminum production, electronics, and semiconductor manufacturing, among other sources [1,2]. The phosphate rock used in fertilizer production contains significant amounts of fluoride, which can be released into wastewater during the production process [3]. The production of aluminum involves the use of cryolite (sodium aluminum fluoride) in the electrolytic reduction process, leading to fluoride emissions in wastewater [4]. Additionally, fluoride compounds are used in etching and cleaning processes in electronics and semiconductor manufacturing, contributing to fluoride-containing wastewater [5]. The excessive concentration of fluoride in aqueous environments poses significant threats to human health and can cause severe ecological damage, which is of substantial concern [6]. According to guidelines established by the World Health Organization (WHO), the permissible limit for fluoride in potable water should not exceed 1.5 mg/L [7,8]. Therefore, managing fluoride levels has become both an environmental and public health imperative, requiring conscientious oversight.

Fluoride removal from drinking water is carried out via adsorption [9], ion exchange [10], and/or reverse osmosis [11]. These technologies are generally a better fit for low concentrations of fluoride. Calcium fluoride precipitation and coagulation is another option for removing fluoride from industrial wastewater to meet discharge requirements and is considered one of the most useful technologies due to its high treatable capacity, simplicity, ease of handling, and low cost [12]. This method is well-suited for the much higher fluoride concentrations typically found in the phosphorus chemical industry and the semiconductor industry. Lime (Ca(OH)_2_) and/or calcium chloride (CaCl_2_) are used to precipitate calcium fluoride (CaF_2_) down to its solubility limit. Calcium fluoride precipitation can reduce the fluoride concentration down to about 10–20 mg/L [13,14], depending on the concentration of total dissolved solids in the wastewater. However, CaF_2_ particles are very fine particles, slow to settle, and difficult to separate [15], which leads to treated effluent still having a substandard fluoride ion concentration and requiring further treatment [5]. Therefore, this process is followed by coagulation to further reduce fluoride levels to meet low discharge limits.

Coagulation is an important process for the removal of suspended particles in wastewater treatment due to its high efficiency, simple operation, and low cost [16]. Coagulants work by neutralizing the electrical charges on particles, which helps reduce the repulsive forces between them. This leads to the formation of small, loose aggregates called microflocs [7,17]. XPS, SEM, zeta potential, and laser particle size measurements have been used to characterize the degree of particle aggregation. Polyaluminum chloride sulfate (PACS) and poly(dimethyldiallylammonium chloride) (PDMDAAC) have been investigated as coagulants for the synergistic treatment of fluoride in graphite industry effluent with Ca(OH)_2_, achieving a fluoride removal rate of 99.8%. Mechanism studies showed that the combination of PACS and PDMDAAC enhanced the synergistic effects of charge neutralization, adsorption bridging, and sweeping [1]. Polyaluminum ferric sulfate (PAFS) has also been applied in the turbidity reduction treatment of coal slurry water, achieving a removal efficiency of over 95% at a dosage of 25 mg/L in the pH range of 6.0–7.0. The turbidity reduction mechanism involves electric neutralization and the adsorption of aluminum and ferric ions [18]. Existing research on the coagulation mechanism has mainly focused on measuring macroscopic physical parameters, the processes of solution microstructure evolution, and the interactions involved in cluster formation, but the reasons for the different charges of clusters have not been sufficiently investigated.

Density functional theory (DFT) and molecular dynamics (MD) are valuable tools for the study of microstructures. DFT calculations can provide insights into the electronic and structural properties of molecules and materials by calculating molecular geometries and bond energies [19]. MD simulations are extensively utilized for studying dynamic processes and behaviors at the molecular level [20]. These simulations enable researchers to investigate complex phenomena such as the movement of atoms and molecules over time, the interactions between molecules, and the dynamic behavior of materials under different conditions of temperature, pressure, and external forces. Molecular dynamics and density functional theory have been used to investigate the mechanisms of phosphate influence on the coagulant effect of ferric sulfate and the flocculant effect of ferric sulfate coagulants, respectively. The results showed that the addition of PO_4_^3−^ can promote the polymerization of PFPS, improve its electric neutralization and adsorption bridging abilities, and finally enhance the coagulation performance [21].

Therefore, this study aimed to investigate the mechanism of ferric sulfate as a coagulant to precipitate soluble fluoride in water at the molecular level. This study simulated the micro-evolution of the aggregation and precipitation of fluoride and calcium ions and the interaction of coagulation molecules with calcium fluoride clusters using MD. The optimization and analysis of the structural properties of hydrated clusters formed during dynamic evolution via DFT revealed the structural evolution of the solution during the precipitation and coagulation process. This study’s findings are expected to provide theoretical support to studies on the mechanism of the ferric sulfate coagulation-assisted precipitation of fluoride ions.

## 2. Results and Discussion

### 2.1. The MD Simulation for Coagulation System

**Figure 1:**
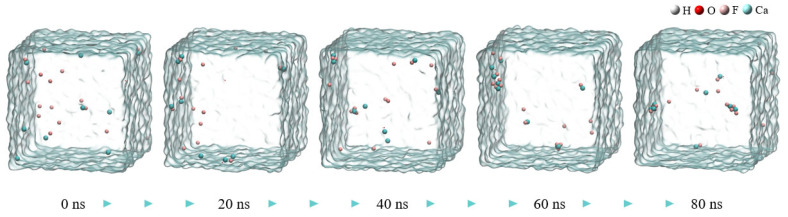
The snapshot MD simulation of CaF_2_ water box.

Figure 1 shows snapshots of the MD simulation of the CaF_2_ water box at different time points. During the aggregation of calcium ions and fluoride ions, small calcium fluoride clusters formed in the box, with some free fluoride ions and calcium ions remaining.

**Figure 2:**
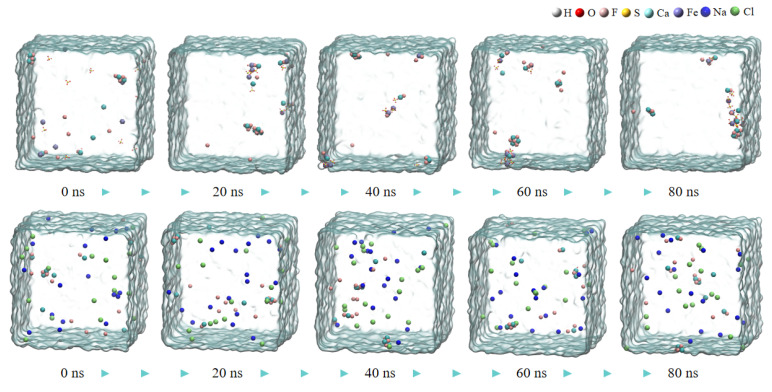
The snapshot MD simulation of CaF_2_ water box with ferric sulfate (the first row) and NaCl (the second row).

The aggregation degrees of particles in the calcium fluoride system at 80 ns in Figure 1 were compared with those in the ferric sulfate system at 40 ns in Figure 2 (the total simulation time for both was 80 ns). There are obviously more free fluoride ions in the calcium fluoride system, and the aggregation degree of the simulated system increases significantly after the addition of ferric sulfate. In comparison to the ferric sulfate system, the sodium chloride system exhibited significantly less obvious aggregation, as shown in Figure 2 (the second row).

**Figure 3:**
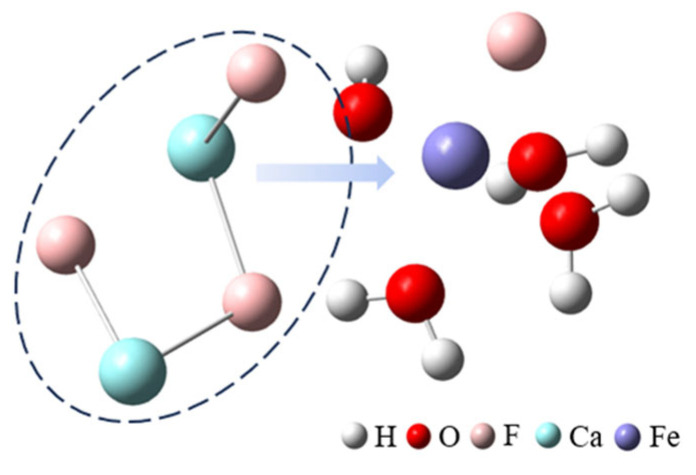
The cluster structure extracted from the MD trajectory.

After adding ferric sulfate, the ferric ions hydrolyze to form hydrated clusters containing hydroxide ions. Then, these hydrated ferric clusters attract small calcium fluoride clusters through the hydroxide ions, resulting in the formation of larger clusters, as shown in Figure 2 (the first row) and Figure 3.

### 2.2. Selection, Optimization, and Analysis of Hydrated Clusters

#### 2.2.1. Selection of Hydrated Clusters

**Figure 4:**
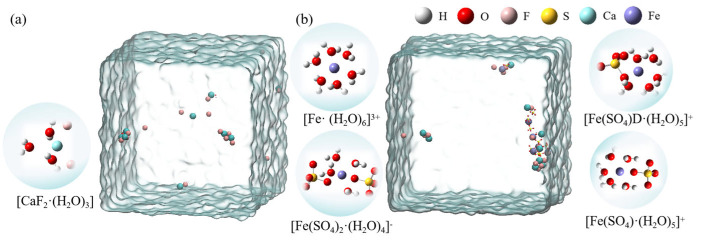
The hydrated clusters constructed in two systems. (**a**) [CaF_2_·(H_2_O)_3_] formed in the CaF_2_ system; (**b**) Ferric ion-related hydrated clusters formed in the CaF_2_ + Ferric sulfate system).

Figure 4 shows the hydrated clusters, which were constructed using detailed coordinate information obtained with VMD after 80 ns of MD simulation for the two systems. Figure 4a shows the hydrated calcium fluoride cluster formed in the CaF_2_ box. Figure 4b shows the hydrated ferric sulfate cluster obtained in the system where ferric sulfate was added as a coagulant. In the ligand clusters formed by a single sulfate ion with ferric ions, the sulfate could coordinate with the ferric ion either as a monodentate ligand or as a bidentate ligand. Therefore, two types of hydrated clusters were named [Fe(SO_4_)·(H_2_O)_5_]^+^ (monodentate ligand) and [Fe(SO_4_)D·(H_2_O)_5_]^+^ (bidentate ligand). Additionally, due to the limited presence of hydroxide ions in the simulated water box, to better illustrate the hydrolysis of ferric ions, we constructed hydrated ferric ion clusters containing various hydroxide ion: [Fe(OH)·(H_2_O)_5_]^2+^, [Fe(OH)_2_·(H_2_O)_4_]^+^, and [Fe(OH)_3_·(H_2_O)_2_].

#### 2.2.2. Optimization of Hydrated Clusters

**Figure 5:**
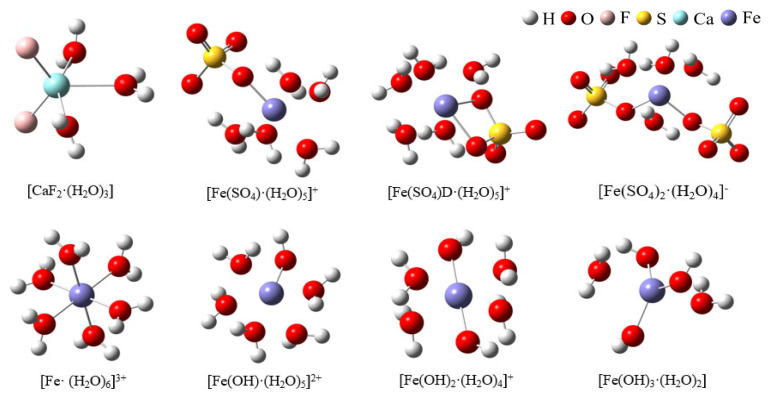
The hydrated clusters optimized at B3LYP/6–311++G(d, p) level.

The selected hydrated clusters were optimized using DFT at the B3LYP/6–311++G(d, p) level, and frequencies were also calculated at this level. The optimized structures are depicted in Figure 5. Table 1 lists the bond lengths between metal ions and ligands in the eight hydrated clusters. According to the data in Table 1, in the hydrated clusters of ferric ions with both sulfate and hydroxide ions, the distance from ferric ions to oxygen in water r(M–O)_H2O_ was generally larger than that from ferric ions to the oxygen of sulfate and hydroxide r(M–O)_OH/SO4_. This indicates that ferric ions have a preference for coordination with oxygen from sulfate ions and hydroxide ions. Additionally, the distance from ferric ions to oxygen in hydroxide r(M–O)_OH_ was smaller than that from ferric ions to oxygen from sulfate ions r(M–O)_SO4_, indicating that ferric ions exhibit a stronger preference for coordination with hydroxide ions.

#### 2.2.3. Analysis of Hydrated Clusters

##### The Binding Energy of Hydrated Clusters

**Figure 6:**
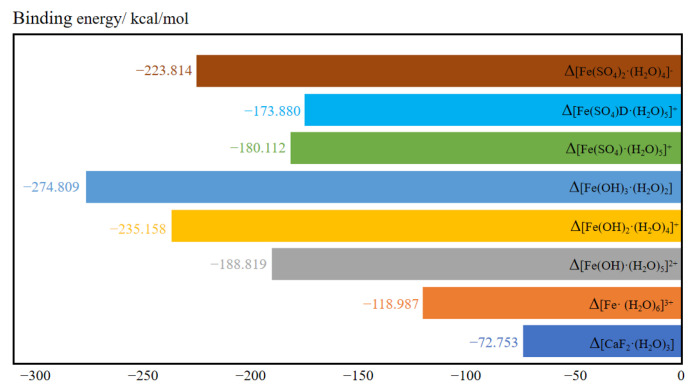
The binding energy of various hydrated clusters.

Figure 6 shows the binding energies of the hydrated clusters. The binding energy of [Fe(SO_4_)D·(H_2_O)_5_]^+^ was greater than that of [Fe(SO_4_)·(H_2_O)_5_]^+^ when one sulfate ion coordinated with a ferric ion, suggesting that the monodentate cluster [Fe(SO_4_)·(H_2_O)_5_]^+^ is more stable than the bidentate cluster [Fe(SO_4_)D·(H_2_O)_5_]^+^. The binding energy of hydrated clusters containing hydroxide was generally lower than that of hydrated clusters containing sulfate when equal numbers of anions coordinated with ferric ions. This indicates that the structure of ferric ions after hydrolysis was more stable than the unhydrolyzed structure. The most stable of these structures is [Fe(OH)_3_·(H_2_O)_2_], which has the lowest binding energy. This also explains why ferric ions readily hydrolyze in aqueous solutions.

##### Topological Analysis

**Figure 7:**
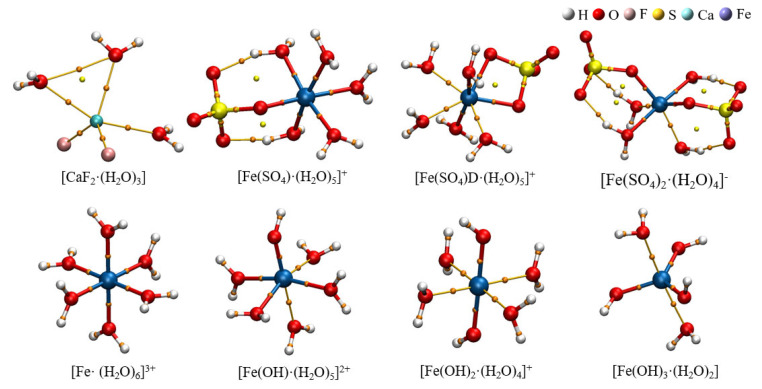
Topology and bond critical point distribution of hydrated clusters.

Figure 7 shows the topology of the electron density of the hydrated clusters. Bond critical points and bond paths were observed between metal ions and water molecules, as well as anions, and they also existed between SO_4_^2−^ ions and water molecules in hydrated clusters of [Fe(SO_4_)·(H_2_O)_5_]^+^, [Fe(SO_4_)D·(H_2_O)_5_]^+^, and [Fe(SO_4_)_2_·(H_2_O)_4_]^−^. It has been shown that the interactions between SO_4_^2−^ ions and water molecules during the formation of the clusters also leads to a decrease in the overall cluster energy, making the structure more stable.

Table 2 shows the mean electron density (ρ), the Laplace value of the mean electron density (∇^2^ρ), and the mean electron energy density (H) at the BCP between the metal ion and the ligand in the hydrated clusters. Among the eight hydrated clusters, the ρ value at the BCPs between the metal ions and water in the [CaF_2_·(H_2_O)_3_] cluster was the smallest. The ρ value at the BCP between the ferric ion and the oxygen in water decreased from 0.07581 to 0.04309 a.u. as the number of hydroxide ions increased from 0 to 3. This indicates that the interaction between a ferric ion and water molecule gradually weakens as the number of hydroxide ions increase in the cluster. When one sulfate ion coordinated with a ferric ion, the ρ value between ferric ions and oxygen atoms in the [Fe(SO_4_)·(H_2_O)_5_]^+^ (monodentate ligand) cluster was greater than that in the [Fe(SO_4_)D·(H_2_O)_5_]^+^ (bidentate ligand) cluster and there were no bond paths between the sulfate and hydrogen in water in the bidentate cluster. This may explain why the [Fe(SO_4_)·(H_2_O)_5_]^+^ cluster has lower energies.

The ∇^2^ρ values at the BCP between the metal ions and the ligands in the hydrated clusters were all greater than zero. This indicates that the interactions of calcium and ferric ions with the ligands were closed-shell interactions, primarily consisting of ionic bonds and van der Waals interactions [22].

In addition, hydrated clusters were formed by other anions with ferric ions, where the average electron densities at the BCP between ferric ions and the oxygen of water were all greater than 0, while the ρ value at the BCP between oxygen in sulfate or hydroxide and ferric ions was less than 0. This suggests partial covalent interactions, which are between ferric ions and inorganic ions in these kinds of clusters. This may be due to the entry of electrons from the anion into the orbital of the ferric ion.

##### Interaction Region Indicator Analysis

**Figure 8:**
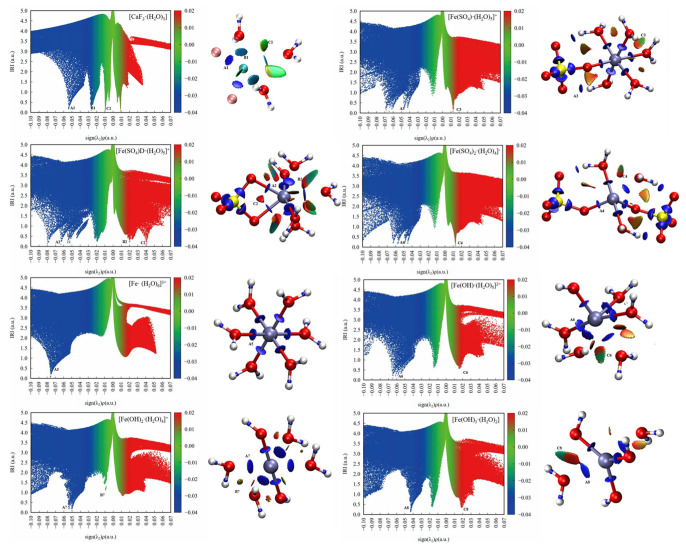
Scatter map between IRI and sign(λ_2_)ρ and isosurface map (IRI = 1.0) of hydrated clusters.

Figure 8 shows scatter maps of the interaction region indicator (IRI) versus the sign(λ_2_)ρ and isosurfaces of eight hydrated clusters. The areas of interaction between the water molecules or anions and the metal ions were generally blue, while the areas of interaction between OH^−^ or SO_4_^2−^ and water molecules were red and partly green. This indicates that the interactions between the metal ions and the ligand or water were strong, whereas the interactions between OH^−^ or SO_4_^2−^ and water molecules were weak van der Waals forces with some steric hindrance.

As shown in the scatter maps of the interaction region indicator (IRI), the point at the bottom of each spike in a scatter map corresponds to an IRI minimum. The spike numbers of the hydrated ferric clusters containing sulfate ions are usually higher than the hydrated ferric clusters containing hydroxide ions, suggesting that there were more interaction sites in the hydrated ferric clusters containing sulfate ions than those containing hydroxide ions, which had less spatial site resistance and were more likely to bind to other molecules.

##### Electrostatic Potential Analysis of Hydrated Clusters

**Figure 9:**
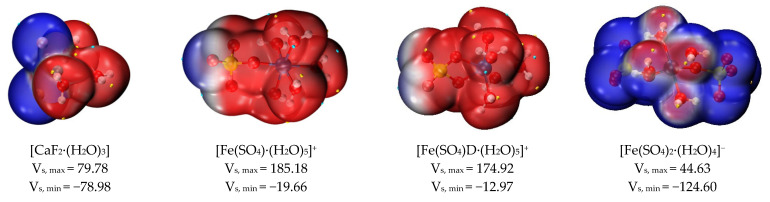

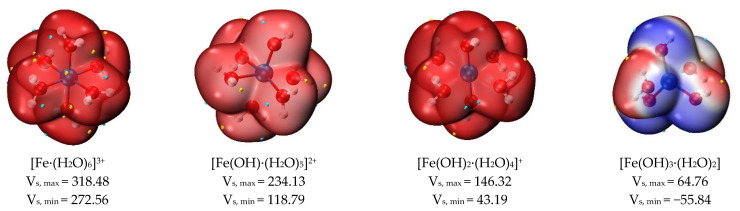
Surface electrostatic potential distribution of hydrated clusters (kcal/mol).

Figure 9 and Table 3 show the values of the surface electrostatic potential and interaction property function of the eight hydrated clusters. The surface electrostatic potential of [CaF_2_·(H_2_O)_3_] varied between −78.98 and 79.77 kcal/mol, and the negative charge was mainly concentrated in the region where the fluoride ions were located. The positive electrostatic potential variance (σ_+_^2^) on the surface of [CaF_2_·(H_2_O)_3_] clusters was smaller than the negative electrostatic variance (σ_−_^2^), indicating that [CaF_2_·(H_2_O)_3_] clusters prefer the negative electrostatic potential part to interact electrostatically with other molecules.

When sulfate and hydroxide ions were used as ligands to form hydrated clusters with ferric ions, and the number of ligands gradually increased, the surface electrostatic potential of these hydrated clusters also gradually decreased. The number of sulfates increases from 0 to 2, and the value of the surface electrostatic potential of the corresponding hydrated clusters decreases, as shown in Figure 9. Due to the positive charge of the ferric ions themselves, the minimum electrostatic potential of the [Fe·(H_2_O)_6_]^3+^ cluster was higher than the maximum electrostatic potential of other hydrated clusters, and the surface electrostatic potential of [Fe·(H_2_O)_6_]^3+^ ranged from 272.56 to 318.28 kcal/mol. In a hydrated ferric cluster containing one sulfate ion, the maximum electrostatic potential of [Fe(SO_4_)·(H_2_O)_5_]^+^ (monodentate ligand) was greater than that of [Fe(SO_4_)D·(H_2_O)_5_]^+^ (bidentate ligand); its minimum electrostatic potential was also smaller than that of [Fe(SO_4_)D·(H_2_O)_5_]^+^ (bidentate ligand). Therefore, the overall electrostatic potential variance (σ_tot_^2^) of [Fe(SO_4_)·(H_2_O)_5_]^+^ (monodentate ligand) was larger than that of [Fe(SO_4_)D·(H_2_O)_5_]^+^ (bidentate ligand), and the degree of surface charge separation (Π) of [Fe(SO_4_)·(H_2_O)_5_]^+^ (monodentate ligand) was larger than that of [Fe(SO_4_)D·(H_2_O)_5_]^+^ (bidentate ligand), as shown in Table 3. Therefore, [Fe(SO_4_)·(H_2_O)_5_]^+^ (monodentate ligand) is more likely to interact with other molecules.

As the pH of the solution rises, the ferric ion gradually hydrolyzes. For the hydrated clusters of ferric ions coordinated with hydroxide ions, as the number of hydroxide ions increased gradually from 0 to 3, the value of Π increased from 11.059 to 24.436. This indicates that the [Fe(OH)_3_·(H_2_O)_2_] cluster is more likely to interact with other clusters via electrostatic potential. In addition, the positive electrostatic potential variance (σ_+_^2^) was greater than the negative electrostatic potential variance (σ_-_^2^), indicating that the [Fe(OH)_3_·(H_2_O)_2_] cluster preferred to use the positive electrostatic potential portion for electrostatic interactions with other clusters. Therefore, during ferric sulfate coagulation, calcium fluoride clusters and ferric hydroxide clusters form larger clusters through electrostatic interaction.

### 2.3. Analysis of Mixed Clusters

**Figure 10:**
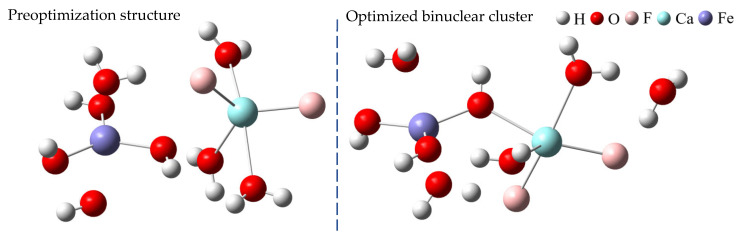
Pre-optimization and optimization of binuclear clusters.

According to the above results, the [Fe(OH)_3_·(H_2_O)_2_] cluster tends to interact with the [CaF_2_·(H_2_O)_3_] cluster. Thus, the structure optimization and frequency calculation of binuclear clusters, which formed after the interaction of calcium fluoride and ferric hydroxide hydrated clusters, were carried out at the B3LYP/6-311++G(d, p) level with the PCM solvent model and the D3 (BJ) dispersion correction. The optimized structure of the binuclear cluster is shown in Figure 10. The two metal centers, Ca and Fe, are connected by a hydroxide ion acting as a bridging ligand. Fluoride ions also form hydrogen bonds with the water molecules of [Fe(OH)_3_·(H_2_O)_2_].

**Figure 11:**
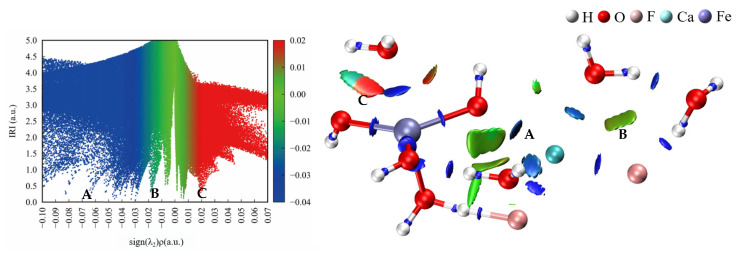
IRI Scatter maps (**left**) and isosurfaces (**right**) of the binuclear cluster.

**Figure 12:**
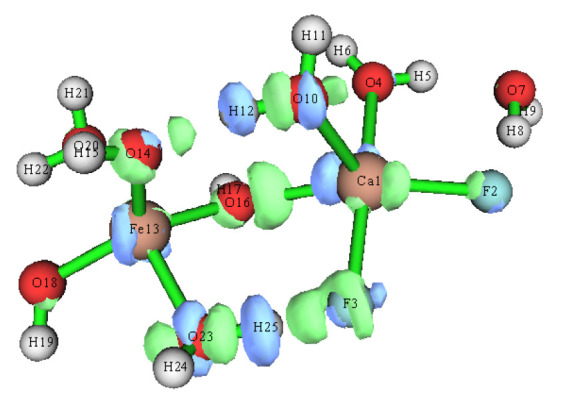
The electron density difference of the binuclear cluster.

Figure 11 shows the IRI scatter plots and isosurfaces of the binuclear cluster. There was a significant electrostatic interaction between Ca^2+^ and OH^−^, as well as between F^−^ and water. Figure 12 shows the change in electron density after the [CaF_2_·(H_2_O)_3_] and [Fe(OH)_3_·(H_2_O)_2_] clusters form binuclear clusters. As shown in the electron density difference diagram, there was a transfer of charge between Ca^2+^ and OH^−^ and between F^−^ and water molecules. This also suggests that calcium fluoride clusters and ferric hydroxide clusters form larger clusters through electrostatic interactions during ferric sulfate coagulation.

## 3. Simulation Details

### 3.1. MD Simulation Details

The microscopic process of CaF_2_ aggregation in aqueous solution and its interaction with ferric sulfate molecules were simulated using molecular dynamics, implemented using the GROMACS 2020.6 package [23]. A cubic water box with a side length of 7 nm, containing calcium and fluoride ions, was constructed. In considering the actual concentration of fluoride ions in phosphogypsum leachate, which typically ranges between 1.5 and 2.0 g/L, and ensuring that the number of ions remained an integer, the concentration of fluoride ions in the simulated water box was set to 0.0774 mol/L (equivalent to 1.47 g/L). As a saturated calcium hydroxide solution has a pH of 12.65, a pH of 12 was chosen for the simulation. Based on the ionization constant of water, approximately two OH^−^ ions were calculated to be present in the 7 nm cubic water box. A simulation of calcium fluoride alone was then conducted for a total duration of 80 ns.

A certain amount of Fe^3+^ and SO_4_^2−^ was added to a CaF_2_ box that was simulated with MD for 40 ns, and this was used as a new system to simulate the coagulation of calcium fluoride with ferric sulfate. Considering the size of the box and the quantity of ferric ions, the concentration of ferric sulfate was set to 0.0145 mol/L. Moreover, in order to compare the influence of ferric sulfate, we chose sodium ions with the same total charge as ferric ions, used chloride ions to balance the charge, and then observed the aggregation degree of the fluoride ions and calcium ions. In order to better observe the aggregation of particles in the system, simulations of 80 ns were carried out on the ferric sulfate system and sodium chloride system. Table 4 shows the number of each type of ion in the simulation box.

The force field used in the calculation process was the GAFF force field [24]. To represent water, the SPC/E model was utilized [25]. The structures of the OH^−^ and SO_4_^2−^ were optimized according to DFT at the B3LYP/6-31+G* level using the Gaussian 16 program [26]. The GAFF force field parameters for all ions involved, based on the Hessian matrix force constants, were generated using the Sobtop code [27]. The RESP (restrained electrostatic potential) charge of the ions was calculated using Multiwfn 3.8 (dev) code [28,29]. In order to avoid the kinetic instability or even collapse of the simulation system due to the excessive force on some atoms at the beginning of the simulation, the maximum interatomic force was set to less than 1000 kJ/(mol·nm), and the energy minimization of the system was performed using the conjugate gradient (CG) method [30]. Temperature and pressure were controlled at a constant of 298.15 K and 1 bar using the V-rescale temperature control method and the Parrinello–Rahman pressure control method [31,32]. Finally, the simulated system simulated molecular dynamics for different time lengths at an equilibrium state of constant temperature and pressure with a time step of 2 fs, and the trajectories of each ion were recorded. The leap-frog method was used to evaluate the dynamics; electrostatic interactions were calculated using the particle mesh Ewald (PME) method; and Van der Waals actions were calculated using the truncation method (with a truncation distance of 1 nm) [33]. Finally, the structure of the relevant hydrated clusters was extracted from the molecular dynamics trajectory and used for the following DFT study.

### 3.2. Structure Optimization of Hydrated Clusters

The structures of the relevant hydrated clusters of Ca^2+^ and Fe^3+^, extracted from the molecular dynamics trajectory file, were optimized based on DFT at the B3LYP/6–311++G(d, p) level, considering weak interactions and solvent effects. The calculations employed D3(BJ) dispersion correction in conjunction with a continuous accessible model (PCM), employing aqueous solution as the solvent system [34]. Their frequencies were calculated at the same level. The structural properties and stability of the hydrated clusters were determined by comparing the variation in bond lengths between the metal ion ligands in different hydrated clusters to analyze the topologies (including the electron density at the critical point, the Laplacian of the electron density, and the energy density, evaluating the binding energy). The electrostatic potential (ESP) of hydrated clusters provides deep insights into the charge distribution and interaction potential of clusters, aiding in understanding the coagulation mechanism. Structural optimization and frequency calculations were performed using the Gaussian 16 software package, and the electrostatic potential of the surface and the interaction characteristic function were calculated using the Multiwfn 3.8(dev) software package.

### 3.3. The Binding Energy for Hydrated Clusters

The binding energy was determined by calculating the energy difference between the energy of each fragment and the whole complex, which was used to determine the stability of molecules [35]. This was calculated as follows:

(1) $$E_{binding}=E\left(AB\right)-E\left(A\right)-E\left(B\right)$$

where $E_{binding}$ is the binding energy, $E\left(AB\right)$ is the sum of electronic and zero-point energies of the complex, $E\left(A\right)$ is the sum of electronic and zero-point energy of fragment A, and $E\left(B\right)$ is the sum of electronic and zero-point energy of fragment B.

### 3.4. Topological and Interaction Region Indicator Analysis

Atoms in molecules (AIM) is a theory that describes bonding in molecules based on the topological nature of the electron density scalar field [36]. The (3, −1) critical point is the critical point where the eigenvalues of the Hessian matrix have two negative and one positive value. This critical point is called the bond critical point (BCP). Topology analysis included the electron density at the critical point (ρ), the Laplace value of the electron density (∇^2^ρ), and the average electron energy density (H).

Electron density (ρ) is closely related to chemical bond strength: the larger the ρ, the stronger the chemical bond, when bonded to combine two elements. The Laplacian function of electron density (∇^2^ρ) is defined as the sum of the three diagonal elements of the Hessian matrix of the electron density [37]. The negative region corresponds to the electron condensation region. It could generally be assumed that a covalent bond is formed if ∇^2^ρ < 0 occurs in the bonding region. Conversely, the interaction between the two atoms could be considered a closed shell-layer interaction if ∇^2^ρ > 0. Energy density is the sum of the kinetic energy density and potential energy density, generally expressed as H(r) or E(r), which represents the energy of an electron at a point. It is considered covalent if this bond has H < 0, whereas H > 0 indicates a non-covalent bond.

The interaction region indicator (IRI) is a visualization method employed to study molecular interactions through graphically representing non-covalent interaction regions, offering visual comprehension of interaction regions within a molecule [38,39]. Essentially, the IRI is the product of the electron density gradient norm and the scaled electron density. The IRI is calculated using the following formula:

(2) $$IRI\left(r\right)=\frac{\left|\nabla \rho \left(r\right)\right|}{[\rho (r){]}^{a}}$$

where a is an adjustable parameter, with the standard definition of IRI using a = 1.1.

IRI analysis is analogous to non-covalent interaction (NCI) analysis, where the sign of the second-largest eigenvalue of the electron density Hessian matrix sign(λ_2_)ρ is mapped onto the IRI isosurfaces, vividly illustrating the nature of the interaction regions. Regions with higher electron density exhibit higher values of sign(λ_2_)ρ, signifying stronger molecular interactions. Conversely, regions with lower electron density correspond to weaker interactions, primarily attributed to weak van der Waals forces. A “weak interaction region” on the scatter plot corresponds to the region where the IRI function value, ∇ρ(r), and ρ(r) are relatively small, and this region is called a spike. The scatter region is then colored according to the IRI value: blue indicates a strong interaction region, green is a weak van der Waals interaction region, and red indicates a spatial repulsion (potential resistance/steric hindrance) interaction region. The colors in the IRI isosurfaces correspond to those of the IRI scatter plot.

### 3.5. Surface Electrostatic Potential Analysis

The electrostatic potential (ESP) at a point in space is the amount of work it takes to move one unit of positive charge from infinity to the point, which is one of the roots of the existence of electrostatic interactions [22]. Electrostatic potentials play a special role in understanding intermolecular interactions, reactive sites and molecular recognition, due to the fact that the intermolecular electrostatic interaction force is the most important long-range interaction force between molecules.

The general interaction properties function (GIPF) of hydrated clusters are analyzed based on the electrostatic potential at the molecular surface. These functions are the overall average (SA) of the electrostatic potential on the van der Waals surface, which is the average of the positive (SA^+^) and negative (SA^−^) regions, and were calculated as follows:

(3) $${\bar{V}}_{S}^{+}=\left(\frac{1}{m}\right)\sum_{i=1}^{m}\;V\left(r_{i}\right)$$

(4) $${\bar{V}}_{S}^{-}=\left(\frac{1}{n}\right)\sum_{j=1}^{n}\;V\left(r_{j}\right)$$

(5) $${\bar{V}}_{S}=\left(\frac{1}{t}\right)\sum_{k=1}^{t}\;V\left(r_{k}\right)$$

where i, j, and k are the electrostatic potentials on the surface of the molecule for positive, negative, and all vertices.

The molecular surface electrostatic potential variance is used to characterize the extent of fluctuations in the cluster surface electrostatic potential. The larger the σ^2^_+_ and σ^2^_-_, the more the system tends to use the positive and negative parts of its electrostatic potential to interact electrostatically with other molecules.

(6) $$\sigma_{+}^{2}=\left(\frac{1}{m}\right)\sum_{i=1}^{m}\;{\left[V\left(r_{i}\right)-{\bar{V}}_{S}^{+}\right]}^{2}$$

(7) $$\sigma_{-}^{2}=\left(\frac{1}{m}\right)\sum_{j=1}^{m}\;{\left[V\left(r_{j}\right)-{\bar{V}}_{S}^{-}\right]}^{2}$$

The average deviation in the electrostatic potential (Π) on the surface of a molecule is a characterization of the degree of charge separation on the surface [40], which is expressed as follows.

(8) $$\Pi =\left(\frac{1}{t}\right)\sum_{k=1}^{t}\;\left[V\left(r_{k}\right)-{\bar{V}}_{S}\right]$$

A larger Π value signifies a higher degree of charge separation, meaning there are more distinct regions of positive and negative charge on the molecule’s surface. Conversely, a smaller Π value indicates a more uniform distribution of charge, with less pronounced charge separation.

### 3.6. Electron Density Difference

The electron density difference is the difference in electron density between two states of a system, typically between the combined system and the sum of its isolated components [41]. In the electron density difference map, green indicates the regions where the electron density increased after applying an electric field, while blue indicates the regions where the electron density decreased after applying an electric field. The electron density difference can help in understanding changes in the distribution of electron density due to chemical interactions, bonding, or external perturbations. Weak interactions are often accompanied by changes in charge, and the graphical representation of the electron density difference between complexes and monomers can help study these charge changes.

## 4. Conclusions

This study systematically investigated the solution microstructure during the ferric sulfate assisted precipitation of calcium fluoride using molecular dynamics simulations and DFT methods. In ferric-containing hydrated clusters, the interactions between ferric ions and ligands are primarily electrostatic interactions and van der Waals interactions. At higher pH levels, ferric ions are easily hydrolyzed to form hydrated clusters of ferric hydroxide. The most stable of these structures is [Fe(OH)_3_·(H_2_O)_2_], which has the lowest binding energy. During the ferric sulfate coagulation process, calcium fluoride clusters and ferric hydroxide clusters form the binuclear clusters through electrostatic interaction. The two metal centers in the binuclear cluster, Ca and Fe, are connected by hydroxide ions. This study reveals the microscale mechanism of fluoride removal by ferric sulfate coagulation through multiscale simulations, emphasizing the critical role of hydroxylated hydrated clusters in coagulation process and offering a theoretical foundation for the advancement of high-efficiency water treatment technologies.

## Figures and Tables

**Table 1:** Distances between calcium and iron ions and different anions and bond angles within anions in clusters.

Cluster	r(M-O)_H2O_	r(M-O/F)_OH/F/SO4_	r(S-O)_SO4_
[CaF_2_·(H_2_O)_3_]	2.481	2.166	-
[Fe·(H_2_O)_6_]^3+^	2.020	-	-
[Fe(OH)·(H_2_O)_5_]^2+^	2.097	1.797	-
[Fe(OH)_2_·(H_2_O)_4_]^+^	2.158	1.858	-
[Fe(OH)_3_·(H_2_O)_2_]	2.258	1.872	-
[Fe(SO_4_)·(H_2_O)_5_]^+^	2.077	1.851	1.518
[Fe(SO_4_)D·(H_2_O)_5_]^+^	2.153	2.045	1.520
[Fe(SO_4_)_2_·(H_2_O)_4_]^−^	2.115	1.910	1.515

**Table 2:** Electron density (ρ), the Laplacian (∇^2^ρ) of electron density, and electron energy density (H) of hydrated clusters/a.u.

Species	BCP	ρ	∇^2^*ρ*	H
[CaF_2_·(H_2_O)_3_]	F…Ca	0.054	0.328	0.007
	O(H_2_O)…Ca	0.026	0.143	0.006
	O(H_2_O)…O(H_2_O)	0.009	0.038	0.001
[Fe(SO_4_)·(H_2_O)_5_]^+^	O(SO_4_)…Fe	0.120	0.638	−0.025
	O(SO_4_)…H(H_2_O)	0.047	0.139	−0.005
[Fe(SO_4_)D·(H_2_O)_5_]^+^	O(H_2_O)…Fe	0.056	0.270	0.001
	O(SO_4_)…Fe	0.079	0.349	−0.006
[Fe(SO_4_)_2_·(H_2_O)_4_]^−^	O(H_2_O)…Fe	0.060	0.287	0.000
	O(SO_4_)…Fe	0.102	0.534	−0.014
	O(SO_4_)…H(H_2_O)	0.047	0.139	−0.005
[Fe (H_2_O)_6_]^3+^	O(H_2_O)…Fe	0.076	0.395	−0.002
[Fe(OH)·(H_2_O)_5_]^2+^	O(OH)…Fe	0.139	0.737	−0.040
	O(H_2_O)…Fe	0.061	0.312	0.001
[Fe(OH)_2_·(H_2_O)_4_]^+^	O(OH)…Fe	0.119	0.631	−0.025
	O(H_2_O)…Fe	0.052	0.253	0.002
[Fe(OH)_3_·(H_2_O)_2_]	O(OH)…Fe	0.116	0.591	−0.023
	O(H_2_O)…Fe	0.043	0.190	0.001

**Table 3:** The values of GIPF based on ESP distribution of clusters on 0. 001 a.u. vdW surface.

Cluster	A/(kcal/mol)	σtot2/(kcal/mol)2	σ+2/(kcal/mol)2	σ−2/(kcal/mol)2	SA/(Bohr^2)	SA^+^/(Bohr^2)	SA^−^/(Bohr^2)
[CaF_2_·(H2O)_3_]	42.304	884.446	349.549	534.897	594.163	348.739	245.424
[Fe·(H_2_O)6]^3+^	11.059	159.353	159.353	0	559.670	559.670	0
[Fe(OH)·(H_2_O)5]^2+^	16.131	480.337	480.337	0	566.476	566.476	0
[Fe(OH)_2_·(H_2_O)_4_]^+^	21.516	631.613	631.613	0	576.554	576.554	0
[Fe(OH)_3_·(H_2_O)_2_]	24.436	498.466	253.745	244.721	530.130	275.057	255.073
[Fe(SO_4_)·(H_2_O)_5_]^+^	55.926	2898.566	2863.488	35.077	713.013	623.128	89.885
[Fe(SO_4_)D·(H_2_O)_5_]^+^	49.611	2390.392	2377.174	13.218	712.552	644.409	68.144
[Fe(SO_4_)_2_·(H_2_O)_4_]^−^	39.227	1294.891	148.075	1146.816	859.964	99.019	760.941

**Table 4:** The number of particles in two kinds of simulation systems.

System	Ca^2+^	F^−^	Fe^3+^/Na^+^	SO_4_^2−^/Cl^−^	OH^−^	H_2_O
CaF_2_	9	16	-	-	2	11,190
CaF_2_ + Ferric sulfate	9	16	6	9	2	10,768
CaF_2_ + NaCl	9	16	18	18	2	10,759

## Data Availability

The data used to support the findings of this study are available from the corresponding author upon request.
